# Pyomyositis as a rare musculoskeletal complication of chickenpox in a pediatric patient case report

**DOI:** 10.1016/j.radcr.2025.06.053

**Published:** 2025-07-15

**Authors:** Mahmoud Mohamad Sbaihat, Ahmad Khaled Othman, Laith Mohammad AlHseinat, Yousef Mohammad Eid Atoom, Faleh Ahmad Al-Sakarneh, Omar Rasmi Al-Tarawneh, Alia Khlaifat

**Affiliations:** a Department of Orthopaedics, Royal Medical Services, Amman 11942, Jordan; b Faculty of Medicine, Yarmouk University, Irbed 21163, Jordan; c Department of Pediatrics, Royal Medical services, Amman 11942, Jordan

**Keywords:** Chickenpox, Pyogenic myositis, *Staphylococcus Aureus*, pus, Musculoskeletal infection, Orthopedic infection

## Abstract

Chickenpox is a common viral infection that mostly affects children from 5 to 9 years old, but it can also occur at any age group. Although typically self-limiting, chickenpox can rarely cause a severe disease and serious complications in approximately 2% of patients. Musculoskeletal complications, though less common, may be both life- and limb-threatening. Clinical manifestations include cellulitis, abscess formation, pyomyositis, necrotizing fasciitis, osteomyelitis, septic arthritis, gangrene, and toxic shock syndrome. Pyomyositis occurs when bacteria enter muscle tissue and start proliferating, resulting in inflammation, pus formation, and possibly muscle abscess. Diagnostic factors in pyomyositis consist of clinical presentation and imaging. Ultrasound (US), scintigraphy, computed tomography (CT), and magnetic resonance imaging (MRI) have been used for diagnostic imaging in suspected cases. Treatment options can be surgical or nonsurgical (e.g., antibiotics) depending on the stage of the disease.

This case was presented to Prince Ali Military Hospital in march 2024, was diagnosed with chickenpox 5 days ago, 5 days after initial presentation, the patient returned with right thigh pain and limping that gradually became more prominent over the past 2 days. The patient's lab results included a white blood cell count (WBC) of 17,400 cells/µL and a positive C-reactive protein (CRP). The MRI showed Rt lateral compartment pyogenic myositis which was drained in theater, admitted to our hospital for 10 days post operatively, she was improved and completely healed.

## Introduction

Chickenpox is a common viral infection that mostly affects children from 5 to 9 years old, but it can also occur at any age group [1]. It is a highly contagious infection caused by varicella zoster virus (VZV). Patients usually present with low-grade fever, general malaise and vesicular rash on multiple areas of the body (eg, face, trunk, limbs) [1]. Chickenpox incidence increases in winter and early spring; as it is transmitted primarily through inhalation of aerosolized respiratory droplets and, secondarily, via direct person-to-person skin contact [1]. According to the World Health Organization, varicella zoster virus (VZV) infects approximately 140 million individuals globally each year, leading to 4.2 million severe cases and approximately 4200 deaths [2].

Although typically self-limiting, chickenpox can rarely cause a severe disease and serious complications in approximately 2% of patients [3]. Particularly in hospitalized, immunocompromised, and unvaccinated patients [2]. The most frequent complications of varicella infection are skin-related and infection-related complications [2]. On the other hand, the least prevalent complications are cardiovascular, genitourinary, and musculoskeletal [2]. Although rare, serious complications with multiorgan involvement can be fatal and should be considered in patients with severe varicella infection [4]. The interval between the onset of varicella symptoms and the development of complications varies. Infectious complications typically arise within 2-6 days following symptom onset [5], whereas other complications, such as neurological manifestations, tend to occur later, with an average onset of 7-16 days after symptoms begin [6].

Musculoskeletal complications, though less common, may be both life- and limb-threatening. Clinical manifestations include cellulitis, abscess formation, pyomyositis, necrotizing fasciitis, osteomyelitis, septic arthritis, gangrene, and toxic shock syndrome [3]. Pyomyositis occurs when bacteria enter muscle tissue and start proliferating, resulting in inflammation, pus formation, and possibly muscle abscess.

Pyomyositis is a bacterial intramuscular infection that progresses through 2 stages: an initial invasive phase marked by a gradual onset of pain and swelling, followed by a suppurative phase characterized by the development of an abscess within or around the affected muscle [7] Diagnostic factors in pyomyositis consist of clinical presentation and imaging. Ultrasound (US), scintigraphy, computed tomography (CT), and magnetic resonance imaging (MRI) have been used for diagnostic imaging in suspected cases [8]. Treatment options can be surgical or nonsurgical (eg, antibiotics) depending on the stage of the disease.

In this report, we present a case of pyomyositis, a rare but serious musculoskeletal complication of chickenpox, which underscores the need for early diagnosis and intervention.

## Case presentation

We report the case of an 11-year-old female with a history of a supracondylar humeral fracture treated 3 years ago with closed reduction and percutaneous pinning (CRPP). She has had no recent surgeries, medical conditions, or hardware removal since then. The patient presented to the emergency department with general vesicular rash. Consequently, she was diagnosed with chickenpox and treated with acetaminophen and anti-histamines to control her symptoms.

Five days after initial presentation, the patient returned with right thigh pain and limping that gradually became more prominent over the past 2 days. Accordingly, she received supportive treatment. However, she presented again 2 days later with fever (39.5°C) and severe right thigh pain with tenderness on the distal and lateral aspect of the thigh. Consequently, she was started on amoxicillin syrup by the pediatric team. The patient's lab results included a white blood cell count (WBC) of 17,400 cells/µL and a positive C-reactive protein (CRP). Additionally, the urine culture grew *Escherichia coli* bacteria the pediatric team agreed to admit the patent and observe her physical status.

**Fig. 1:**
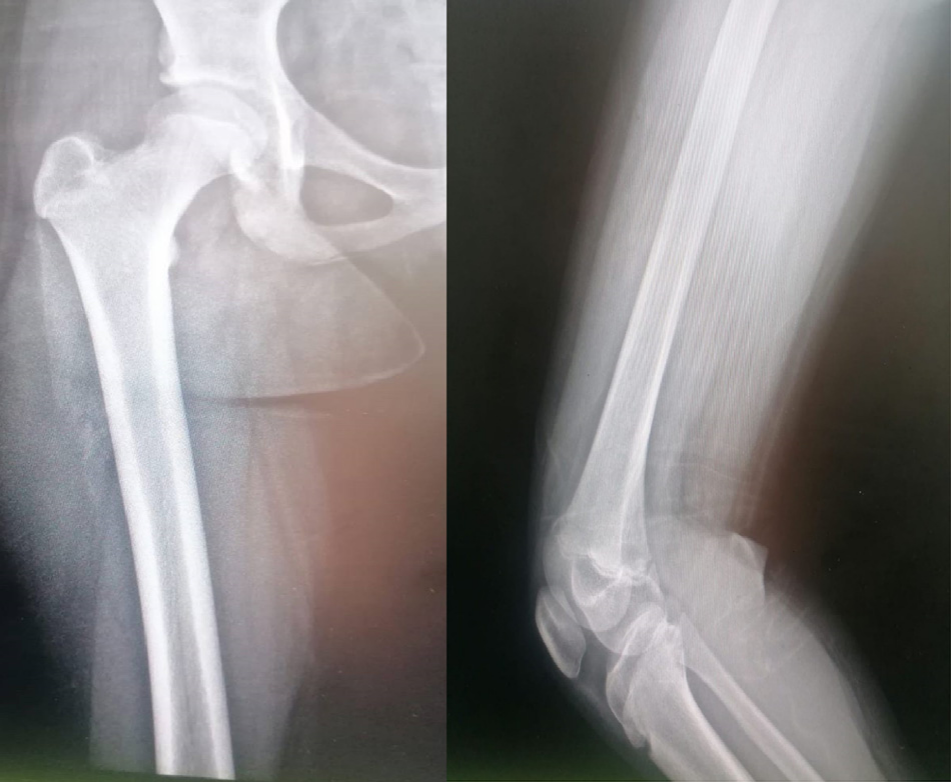
Initial presentation x-ray AP and lateral views showing no obvious pathology.

On the next day, the patient reported no improvement in her symptoms despite antibiotic treatment. Thus, the orthopedic team was consulted about the possibility of septic knee or osteomyelitis. Examination of the patient’s right lower limb revealed erythema and hotness on the lateral aspect of distal thigh, inability to bear weight, decreased range of motion in the knee, and distal femur tenderness. A plain radiograph of the knee was grossly normal (Fig. 1). However, ultrasound (US) imaging of the knee showed subcutaneous edema without joint effusion.

A day later, the patient’s lab results contained a (WBC) of 19,000 cells/µL, an erythrocyte sedimentation rate (ESR) of 80 mm/h, and a positive (CRP). Aspiration of the knee and synovial fluid analysis revealed a (WBC) of 26,000 cells/µL and red blood cell count (RBC) of 45,000 cells/µL. The orthopedic team concluded that there is no evidence of septic arthritis with a recommendation of obtaining an MRI of the knee and femur, along with admission to the pediatric ward.

**Fig. 2:**
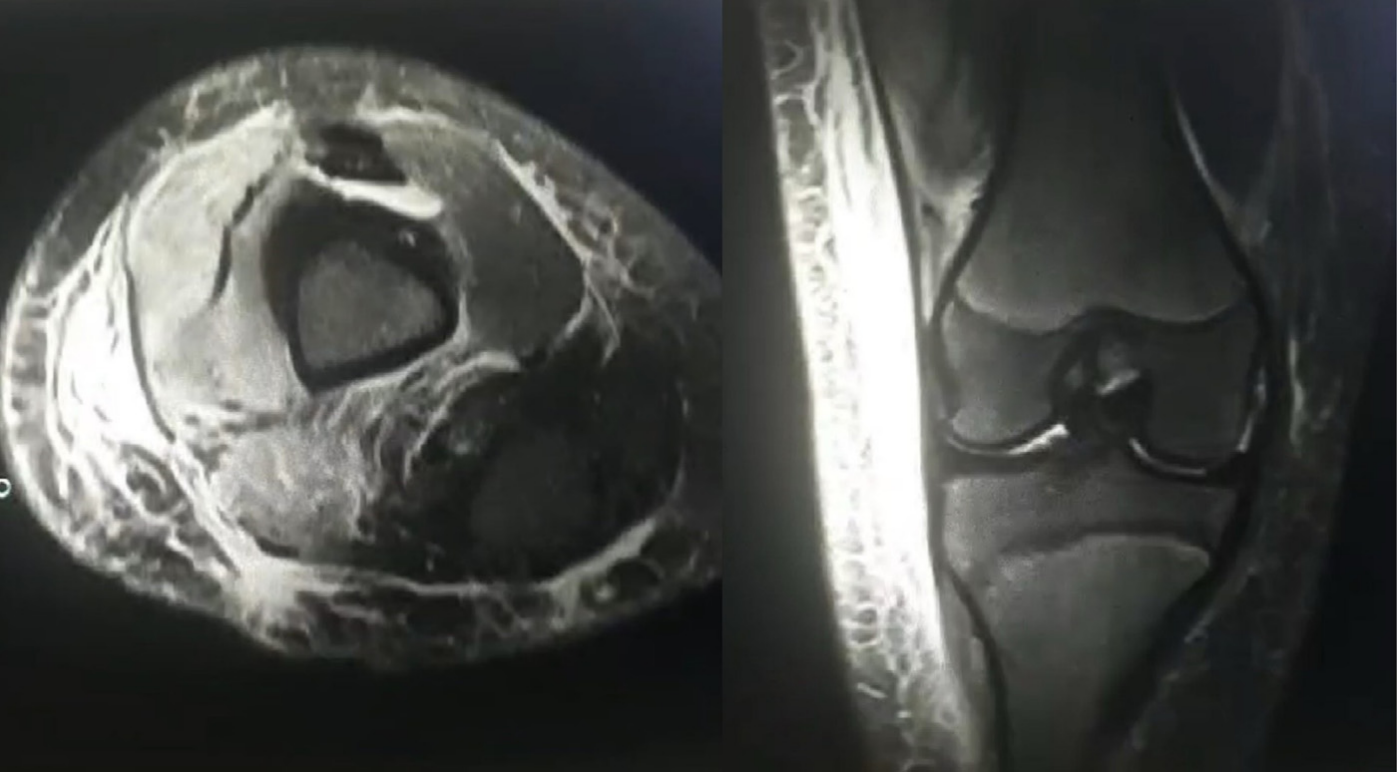
Axial and coronal MRI sections showing significant subcutaneous and muscle edema indicating myositis with no intramuscular collection.

On the following day, the patient’s lab results showed a (WBC) of 21,500 cells/µL. Magnetic resonance imaging (MRI) of the right knee revealed mild joint effusion and soft tissue edema, suggesting inflammation around the knee (Fig. 2). The pediatric team discharged the patient and advised that she going to be followed closely in the outpatient clinic even she still has high WBC count.

**Fig. 3:**
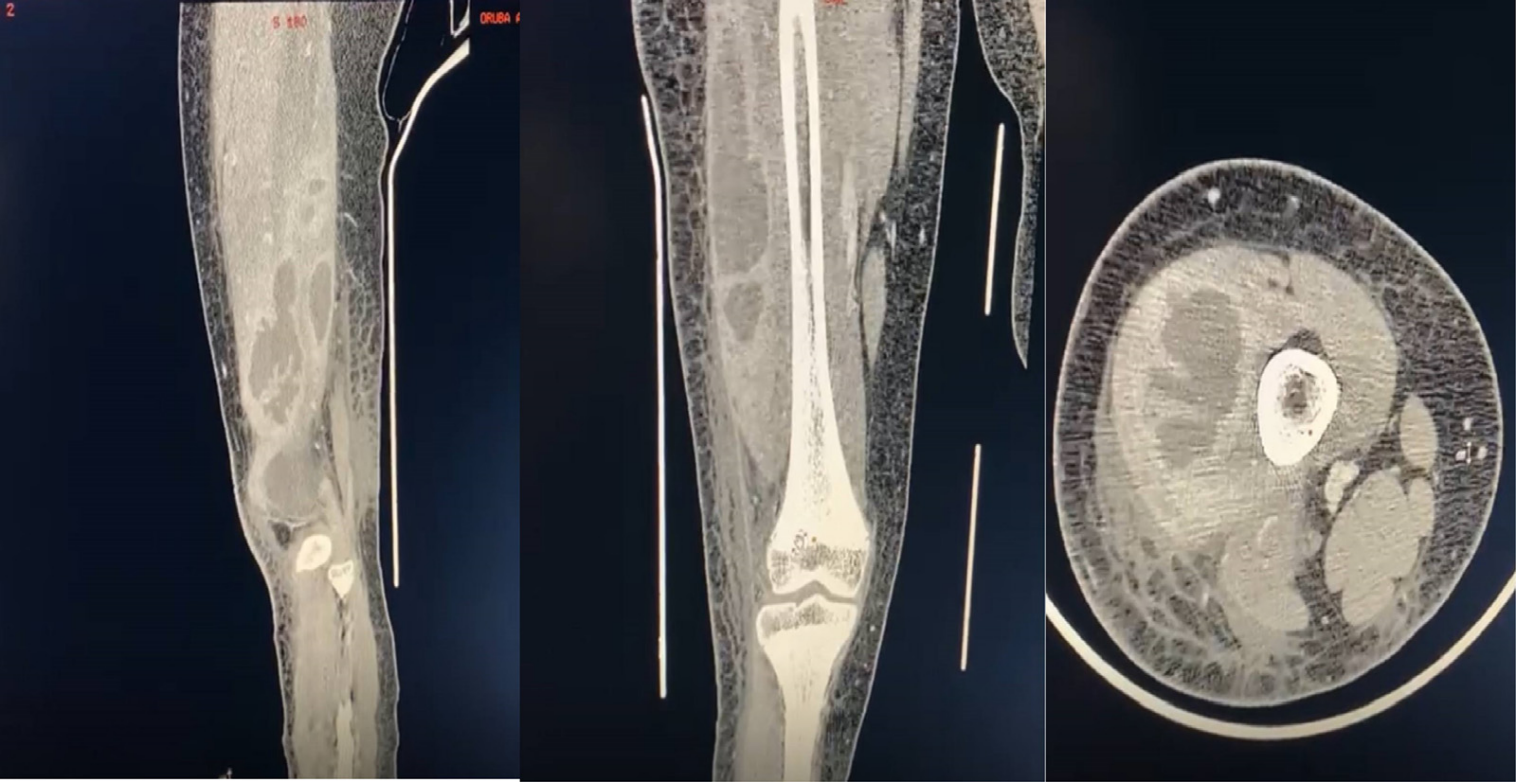
Sagittal, coronal and axial CT scan of Rt thigh showing intramuscular multilobulated collection.

The patient continued to suffer from the same symptoms in the next days, after which she presented to emergency department with the same complain associated with high grade fever. On that day, the patient’s lab results showed a (WBC) of 29,000 cells/µL, an (ESR) of 130 mm/h, and a positive (CRP). With the lab results remaining high, an ultrasound (US) and computed tomography (CT) of the right thigh were obtained. Imaging revealed a well-defined, thick collection located intramuscularly, measuring 5.3 × 2.2 cm, superior to the right knee over the lateral compartment, with no evidence of joint effusion (Fig. 3). Thus, the patient was admitted to the orthopedic ward and scheduled for a surgery to incise and drain (I/D) the affected area the next day.

**Fig. 4:**
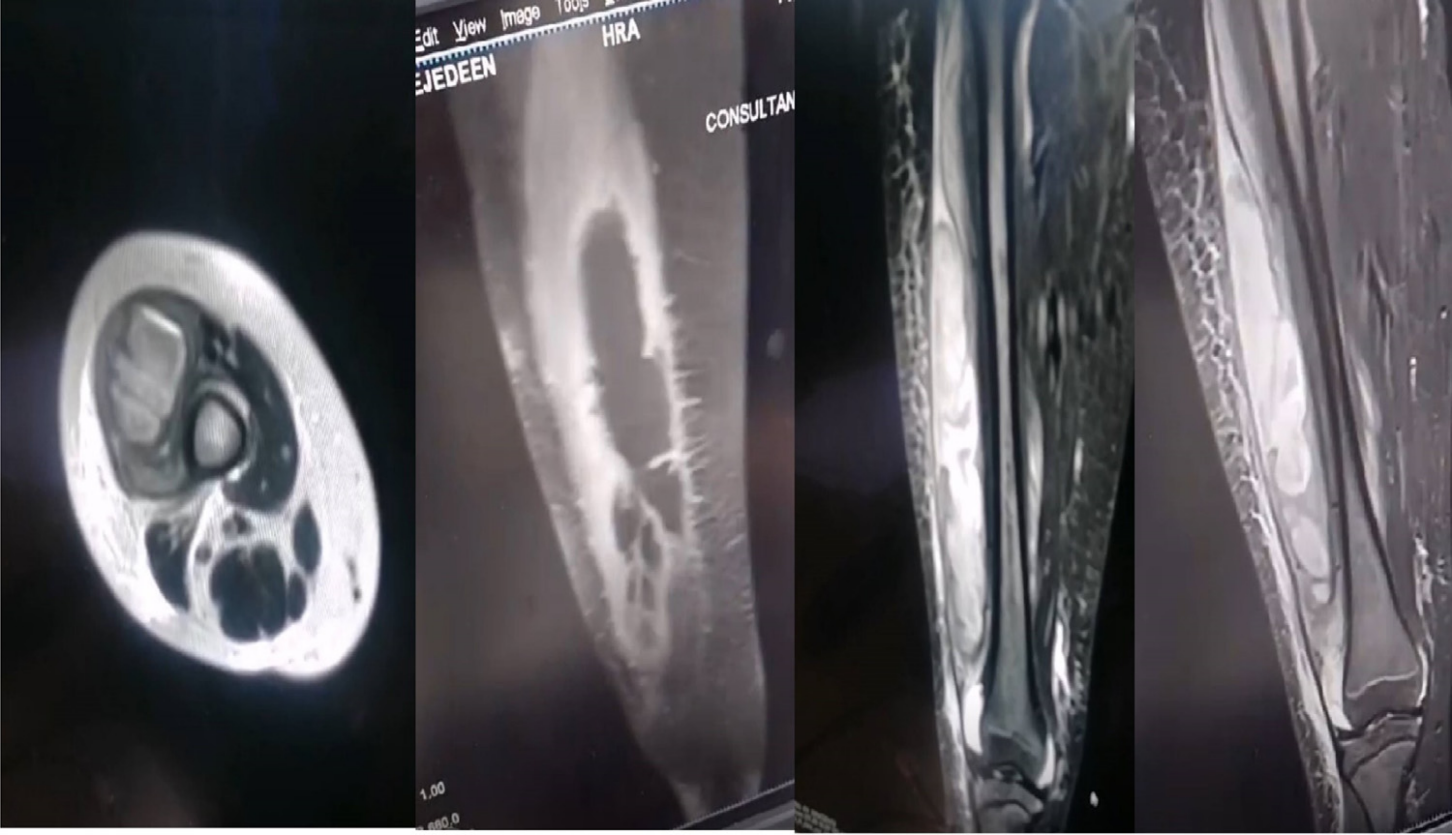
Axial, sagittal and coronal sections of Right thigh MRI showing significant amount of deep Intramuscular collection with no obvious signs of osteomyelitis.

**Fig. 5:**
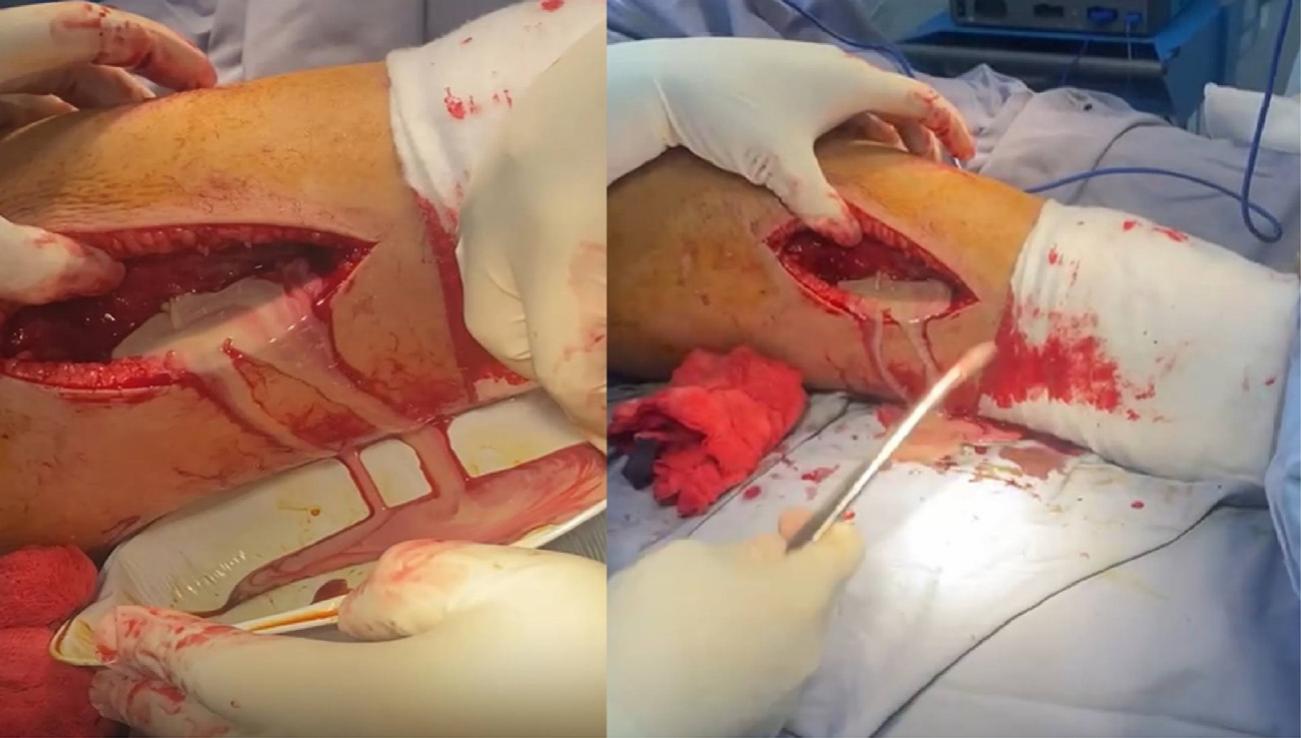
Intraoperative clinical photos showing significant amount of Frank pus was drained from lateral compartment of right thigh.

Preoperatively, the patient’s lab results showed a (WBC) of 35,000 cells/µL and a (CRP) of 241 mg/L. Magnetic resonance imaging (MRI) of the right lower limb revealed a deep collection of fluid in the lateral compartment of the thigh deep to the fascia with proximal leg subcutaneous edema with no obvious signs of osteomyelitis (Fig. 4). During the operation, lateral approach to the thigh was used, a collection of frank pus was present in the vastus lateralis muscle and the lateral compartment (Fig. 5), large amount of fluid was used for irrigation and necrotic tissue was excised, drain was applied and followed regular till clinically improved. Culture of soft tissue was taken intraoperatively grew *staphylococcus epidermidis*. Although *S epidermidis* infections are more commonly associated with postsurgical cases, the patient has had no recent surgeries, medical conditions, or hardware removal.

**Fig. 6:**
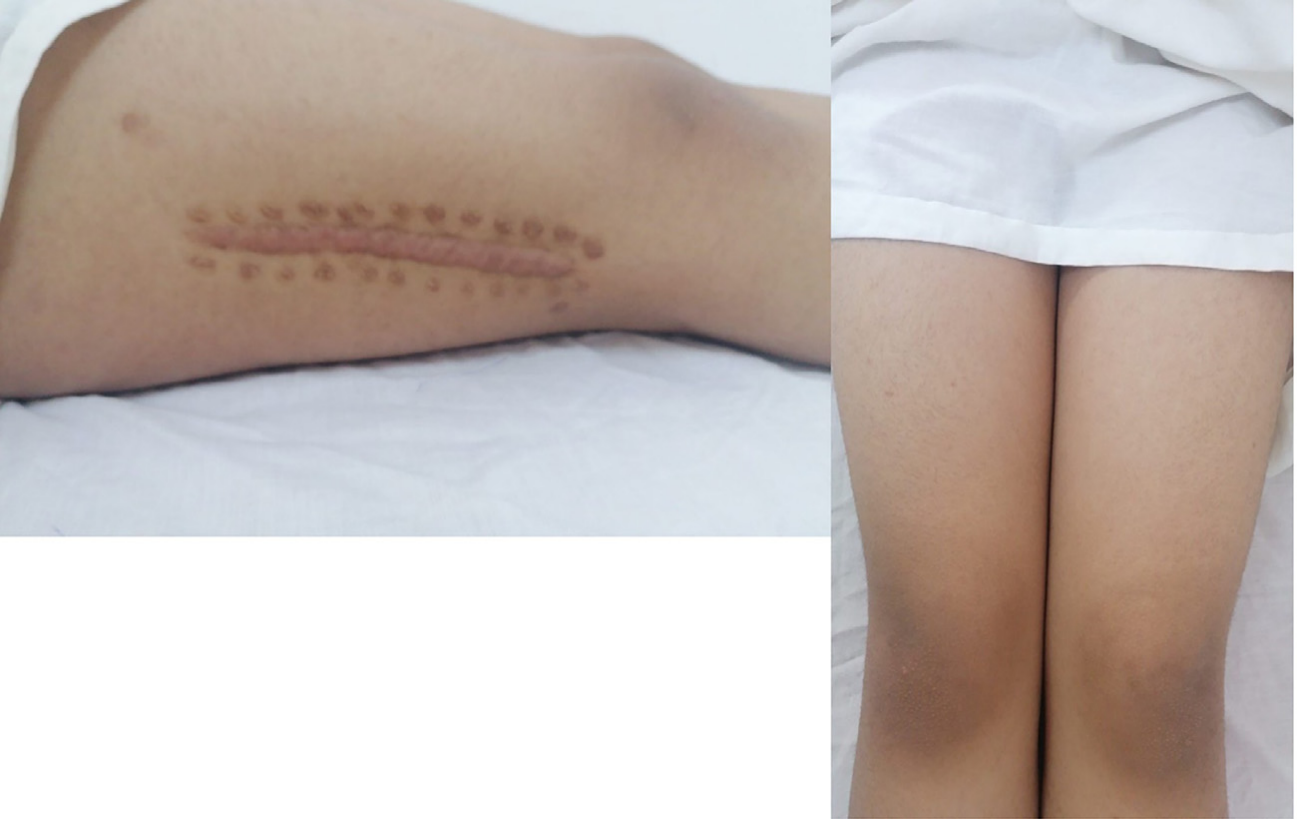
Clinical photos showing healed SSI and symmetrical thighs with excellent function outcomes.

Follow-up of the patient demonstrated an improvement in her symptoms, with a return of function, healed SSI, increased range of motion (ROM), and a reduction in both (WBC) and inflammatory markers till she returns back to her normal function without serious complication like septic arthritis, osteomyelitis and necrotizing fasciitis (Fig. 6).

## Discussion

Chickenpox is usually a self-limiting infection affecting young patients in the pediatric age group. It is often characterized by low grade fever, malaise, an itchy and vesicular rash [1]. The onset of symptoms is typically 11-20 days following the exposure to varicella zoster virus [1]. Additional symptoms may include lowered urine output, dehydration, nausea, muscle aches, loss of appetite, and headaches [1]. Although most chickenpox diagnoses are made clinically, the virus can also be identified through molecular techniques such as PCR or by isolating it from fluid or cells taken from the base of a vesicular lesion [9].

While chickenpox is generally self-limiting, treatment with anti-viral medications such as acyclovir may be beneficial to reduce the severity and shorten the duration of symptoms if given within 24 hours of the onset of infection [9]. However, complications following varicella infection can still occur, these commonly include severe varicella and skin-related and infection-related complications (ie, balanitis, cellulitis, hemorrhagic vesicular rash, urticaria, skin or soft tissue infection) [2]. Less commonly, musculoskeletal complications account for approximately 6% of all chickenpox complications and include cellulitis, abscess, pyomyositis, necrotizing fasciitis, osteomyelitis, septic arthritis, gangrene, and toxic shock syndrome [3]. While septic arthritis and osteomyelitis are common complications of varicella, the patient's persistent localized muscle pain, along with imaging findings, pointed towards pyomyositis.

Pyomyositis presentation typically has 3 stages [10]. Firstly, the invasive stage consists of inflammation and pain of the affected muscle and may be associated with mild leukocytosis. In this stage, examination often reveals firm muscles without signs of fluctuation, a palpable abscess, or overlying erythema [10]. Secondly, the suppurative phase includes formation of muscle abscess with patients reporting intense pain, increasing swelling, and fever. Most cases are diagnosed during this stage, which typically spans 1 to 3 weeks [10]. In the third stage, systemic features become prominent, including toxicity, septicemia, shock, and formation of multifocal abscesses [10]. It can be a serious complication leading to a severe disease and even death [10]. It is most commonly caused by *Staphylococcus aureus* (*S. aureus*) [10], but can be caused by other infectious agents such a *S epidermidis* (*S. epidermidis*), which is the causative agent in this case report.

Pyomyositis poses a diagnostic challenge due to its nonspecific clinical presentation and its relatively rare occurrence. A combination of recognition of the presenting symptoms and imaging investigations is needed to reach a definitive diagnosis. Ultrasound (US), scintigraphy, computed tomography (CT), and magnetic resonance imaging (MRI) have been used for diagnostic imaging [8]. Imaging is especially beneficial to exclude differential diagnoses of pyomyositis such as osteomyelitis and septic arthritis. Ultrasound should be the first-line imaging modality for evaluating the extremities. Ultrasound-guided biopsy or aspiration can be helpful to identify the causative organism. Magnetic resonance imaging (MRI) is essential for detecting pyomyositis in the pelvic region and for distinguishing it from osteomyelitis [8]. In this case report, ultrasound was instrumental in detecting subcutaneous edema, while CT revealed a thick multilobulated collection, crucial in diagnosing pyomyositis.

Successful management of pyomyositis depends on early recognition and initiation of appropriate measures including careful cardiothoracic evaluation, antibiotic therapy, incision and drainage, and subsequent rehabilitation of the patient [11]. In early stages, conservative treatment with antibiotics alone or antibiotics and percutaneous aspiration can be sufficient [10] Antibiotics with anti-staphylococcal and anti-streptococcal coverage can be recommended to treat pyomyositis, with modifications depending on local epidemiology and resistance patterns [12] Antibiotic treatment for 3-4 weeks is generally adequate, starting via intravenous route (IV) and switching to an oral antibiotic (PO) promptly [12] In later stages, surgical decompression is required in 50% of cases [10]. In this case report, both treatment options with antibiotics (i.e., amoxicillin) and surgical incision and drainage were needed to achieve full recovery.

Although *S aureus* is the most common pathogen responsible for pyomyositis, *S epidermidis* was identified in this patient, which may be linked to its presence in the body’s normal flora and its increased resistance to antibiotics.

## Conclusion

Pyomyositis is a rare complication of chickenpox in children with a possibility to cause severe or even life-threatening illness. Prompt diagnosis with clinical evaluation and imaging studies in addition to early initiation of treatment with antibiotics and surgical interventions are crucial for more favorable outcomes and a complete recovery. Although *S aureus* is the most frequent causative agent in pyomyositis, other agents such as *S epidermidis* should be considered to determine the most appropriate treatment.

## Patient consent

Written informed consent was obtained from the patient for publication of this case report and accompanying images. written consent is available for review by the Editor-in Chief of this journal on request.
